# Virtual Care and the Inverse Care Law: Implications for Policy, Practice, Research, Public and Patients

**DOI:** 10.3390/ijerph191710591

**Published:** 2022-08-25

**Authors:** Hassane Alami, Pascale Lehoux, Sara E. Shaw, Chrysanthi Papoutsi, Sarah Rybczynska-Bunt, Jean-Paul Fortin

**Affiliations:** 1 Nuffield Department of Primary Care Health Sciences, University of Oxford, Radcliffe Observatory Quarter, Woodstock Road, Oxford OX2 6GG, UK; 2 Center for Public Health Research and Department of Health Management, Evaluation and Policy, University of Montreal, Montreal, QC H3C 3J7, Canada; 3 Community and Primary Care Research Group, Faculty of Health, Plymouth University, Plymouth PL6 8BX, UK; 4 VITAM Research Centre on Sustainable Health, Faculty of Medicine, Laval University, Quebec, QC G1J 2G1, Canada

**Keywords:** virtual care, inverse care law, determinant of health, direct-to-consumer, telehealth, digital health, health policy, equity, capabilities, COVID-19

## Abstract

Virtual care spread rapidly at the outbreak of the COVID-19 pandemic. Restricting in-person contact contributed to reducing the spread of infection and saved lives. However, the benefits of virtual care were not evenly distributed within and across social groups, and existing inequalities became exacerbated for those unable to fully access to, or benefit from virtual services. This “perspective” paper discusses the extent to which challenges in virtual care access and use in the context of COVID-19 follow the Inverse Care Law. The latter stipulates that the availability and quality of health care is inversely proportionate to the level of population health needs. We highlight the inequalities affecting some disadvantaged populations’ access to, and use of public and private virtual care, and contrast this with a utopian vision of technology as the “solution to everything”. In public and universal health systems, the Inverse Care Law may manifests itself in access issues, capacity, and/or lack of perceived benefit to use digital technologies, as well as in data poverty. For commercial “Direct-To-Consumer” services, all of the above may be encouraged via a consumerist (i.e., profit-oriented) approach, limited and episodic services, or the use of low direct cost platforms. With virtual care rapidly growing, we set out ways forward for policy, practice, and research to ensure virtual care benefits for everyone, which include: (1) pay more attention to “capabilities” supporting access and use of virtual care; (2) consider digital technologies as a basic human right that should be automatically taken into account, not only in health policies, but also in social policies; (3) take more seriously the impact of the digital economy on equity, notably through a greater state involvement in co-constructing “public health value” through innovation; and (4) reconsider the dominant digital innovation research paradigm to better recognize the contexts, factors, and conditions that influence access to and use of virtual care by different groups.

## 1. Introduction

Over 50 years ago, Julian Tudor Hart proposed the Inverse Care Law, suggesting that the availability and quality of health care is inversely proportionate to the level of population health needs [1,2,3]. This is particularly the case when health care services are subject to market forces that negatively influence the services available to disadvantaged populations [2,4]. The latter refer to all individuals or groups who lack just and fair opportunities and face barriers to health “such as poverty, discrimination (e.g., ethnicity, culture, gender, age, geography), and their consequences, including powerlessness and lack of access to good jobs with fair pay, quality education and housing, safe environments, and health care” [5]. In this context, only people who can afford to pay have access to private health care services [2,4]. The Inverse Care Law also manifests itself in public, or so-called universal health systems, where providers are largely distributed according to the size of the population in different geographical areas rather than needs [6]. In some areas, this can result in long waiting lists and shorter consultation times despite higher rates of co-morbidities and complex needs (e.g., chronic diseases, psychosocial problems) [2,7]. These communities face poorer health outcomes, shorter life expectancy and earlier death, and receive reduced quality and quantity of health care services despite the presence of risk factors (e.g., inadequate housing, food scarcity, physical labor, poverty) [8,9]. The Inverse Care Law remains relevant today. However, few public policies are in place to successfully redress its effects and support access to health care services, especially for disadvantaged populations [6].

During the COVID-19 pandemic, access to health care was restricted due to physical distancing requirements, leading to increased use of virtual care [10,11,12]. The latter refers to patient–clinician (e.g., physician, nurse, allied care professionals) “interactions related to diagnosis, evaluation, and management conducted remotely using some combination of text, audio, and video either synchronously or asynchronously” [13]. Virtual care has been a key channel for health care services provision in many health systems [10,11,12,14,15] (Box 1). This rapid deployment of virtual care, whether publicly reimbursed or sold by commercial platforms, has raised concerns regarding equity and the exacerbation of systemic and structural inequalities in accessing and using health care services [10,16]. According to the World Health Organization (WHO), equity is defined as: “the absence of avoidable, unfair, or remediable differences among groups of people, whether those groups are defined socially, economically, demographically or geographically or by other means of stratification” [17]. During the pandemic, physical distancing and containment directives may have limited the ability of many disadvantaged populations to access and use health care services, likely resulting in poor disease monitoring and care management and/or delays in diagnosis and treatment [18,19]. For example, in Quebec (Canada), by mid-April 2020, consultations due to heart attacks had decreased by 40–60% and by 80% for certain vascular events (e.g., minor strokes, transient ischemic attacks) [19]. Similar results were observed in oncology where more than 4000 people with cancer were not diagnosed between March and June 2020. Comparable findings were reported in mental health and addiction services [19].

Box 1 **Examples of virtual care deployment around the world at the onset of the COVID-19 pandemic.**

In the United Kingdom (UK), remote consultations were introduced at the onset of the pandemic in March 2020, with the government later announcing the “remote by default” policy would remain “even after the pandemic had receded” (although this was subsequently reversed) [20,21].
Over this period, the UK media mainly presented virtual care in active terms as “the agents of change” able to increase efficiency and effectiveness [21]. It also emphasized the urgency of change within the National Health Service (NHS) and the role of private entrepreneurship in delivering virtual care solutions and services.
In North America, the political and media space was dominated, at least at the beginning, by an optimistic and enthusiastic discourse on virtual care “that will transform/revolutionize healthcare delivery” [10,11,22,23,24,25].
In China, virtual care proved vital as the government banned in-person interactions during lockdowns [26].
In South Africa, it has also taken an important place in the provision of health care services, but quickly raised concerns, particularly with regard to significant variation between urban centres and rural, remote or resource-poor areas [27].
Over the same period, Australia pressed “fast-forward” to ensure its virtual care capabilities support the response to COVID-19 [28,29]. The government provided exceptional funding for the deployment of a “new Medicare service” with universal virtual care, accessible “at no cost” for patients from their homes [28,29].

Pre-pandemic literature raised concerns about the social impacts of virtual care [30,31,32,33,34,35,36] and “digital exclusion”, which refers to unequal access to digital technologies and lack of skills to use them [37]. To date, this (largely social science-based) literature has largely been side-lined or ignored, with limited attention on the part of decision-makers and technology promoters on the potential of technologies to exacerbate health inequalities [38,39,40,41,42,43]. According to Gann (2019), “as healthcare systems progress their digital transformation agenda, there is a real danger of a new digital Inverse Care Law whereby those citizens most in need of accessing new digital services will be left behind again, due to their lack of digital skills and access” [41]. In the same vein, Davis et al. (2021) highlight that if this challenge is not acknowledged and addressed seriously, it would mean that virtual care may, inadvertently, further widen health inequities, “thus integrating the Inverse Care Law into the digital era as a digital Inverse Care Law” [38]. Therefore, issues of equity and the digital divide, exemplified by the Inverse Care Law, should have a central place in the public debate. The digital divide underscores a complex range of individuals and groups whose level of technology access, adoption, and use varies over time under the influence of socio-political, economic, historical, and cultural contexts; infrastructure; as well as individual characteristics such as education, disability, gender, or age [44]. People who were on the “wrong side” of the digital divide were most likely to have higher comorbidities, more severe symptoms, and higher mortality rates in the context of COVID-19 [44].

This “perspective” paper discusses the extent to which access and use issues relating to virtual care during the COVID-19 pandemic follow the Inverse Care Law. In doing so, we aim to provide one of the first in-depth analyses of the Inverse Care Law in the context of virtual care. Informed by the current international literature on virtual care in the context of COVID-19 and beyond, we mainly draw on our own research on digital health (in Canada and the UK) to reflect particularly on virtual care and disadvantaged populations. The ways in which health care and virtual care are delivered are shaped by a range of factors and conditions. In the following sections, we therefore set out how the Inverse Care Law is shaped by, firstly, virtual care provided and/or reimbursed by public and universal health systems (theoretically accessible and usable by all citizens according to their needs and not their ability to pay) and, secondly, virtual care provided by commercial platforms (“Direct-To-Consumer”, accessible based on ability to pay).

We conclude that in some situations, virtual care provided by both private and public health systems contributes to increased inequalities in access to and use of health care services. Finally, we set out some implications for policy, practice, research, the public, and patients in order to address the Inverse Care Law in relation to virtual care and the equity concerns that underlie it.

## 2. How Does the Inverse Care Law Manifest Itself in Virtual Care Reimbursed by the Health System?

At the onset of the COVID-19 crisis, virtual care was widely adopted to provide health care services to the population in many health systems [10,45,46,47]. In some cases, the “digital-first” model was privileged for scheduling appointments or for the first clinical consultation (e.g., total virtual triage) [48,49]. Some private virtual services, mainly “virtual walk-in clinics”, historically directly paid by individuals (“out of pocket”), were covered and reimbursed by public and universal health systems if patients had public health insurance [50,51,52]. In many health systems, digital technologies, video- (e.g., teleconsultation platforms) or audio-based (e.g., telephone), became a prerequisite for access to health care services [10,53,54].

In this (albeit extraordinary) set of circumstances, people from certain disadvantaged populations appeared to be in the blind spot of decision-makers [10,45,46,55], resulting in structural and systemic barriers to accessing health care services. For example, people with limited digital access and literacy are less likely to use patient portals and digital health technologies in normal circumstances [16]. It is difficult for them to be “virtually” cared for and followed over time [56]. During the pandemic, certain health organizations required patients to register on a patient portal (or app) or complete an online form (e.g., providing phone number or email address for teleconsultation purposes) to use services. Such digital requirements present an additional barrier for some people.

Most virtual care technologies in use were developed before the crisis. With some exceptions (e.g., Scotland) [57,58], the approach adopted was mainly technology-driven, with limited involvement of, or co-design with the different groups and/or communities for whom such services were intended [33,59,60,61]. As technology became the main gateway to the health system during the pandemic, shifting to virtual care replicated and/or strengthened existing inequalities [16]. In the sections below, we set out how, for certain disadvantaged populations, the Inverse Care Law was exacerbated as technology became an additional obstacle to other constraints that people faced before the pandemic. The result is that the comparative advantages of virtual care are concentrated on populations able to maximize its technological benefits [44].

### 2.1. Capacity to Access Digital Technologies

Some people may not have access to high-speed internet, suitable hardware and software, and/or a private space to conduct a confidential virtual consultation [62]. The use of virtual care requires the presence of, and access to good quality bandwidth [46,63]. For example, in Canada, only a quarter of the country’s indigenous communities have access to high-speed internet, compared to 97% in urban areas [64]. In Montreal, up to 30% of the residents in neighborhoods that registered a greater mortality due to the COVID-19, do not have an e-mail address [10]. In the UK, some disadvantaged populations spend up to half of their family expenses on telephone/internet subscriptions [38]. Using primarily “pay-as-you-go” contracts, rather than monthly subscriptions, these populations face inherent barriers to using limited data [8,65]. Lacking quality equipment and connectivity, they were more likely to be consulted by telephone. Studies have reported that telephone may be sub-optimal compared to video or in-person consultations (e.g., visual cues and body language, medical errors, diagnosis accuracy, readmission rates) [66,67,68,69].

### 2.2. Capacity and Propensity to Use Digital Technologies

Inequalities are not only shaped by the difficulty of access to technology, but also by variations in the willingness and capacity to use it [70]. As a result, access does not guarantee use. Some populations may not be aware of the existence of virtual care or may lack the digital and clinical skills to use them, especially internet-based services [10,71,72]. For example, the UK Office for National Statistics 2020 data showed that 6.3% (≈4.2 million people) of UK adults have never used the internet, including 19% (≈2.6 million people) of disabled adults. Among individuals over 75 years old, 54% are “recent internet users” [39,73]. While low literacy is recognized as a major barrier to service use [74], people who do not speak the official language of the country where they live had less access to virtual care during the pandemic [10,55]. Medical interpreter services were not always available, largely due to the resources and time required, as well as the additional complexity it brought to digital interactions [75]. In addition, the literature has reported that some vulnerable people, particularly those with limited literacy, may require up to twice the time to complete a task on their electronic health record (EHR) and are more likely to misinterpret the information provided (e.g., treatment plan) [76,77]. As a result, clinicians under pressure might prioritize patients with the ability to use the technology [30,78]. Such attitudes on the part of a clinician could also be explained by the lack of pre-pandemic training on virtual care, and on ways to manage vulnerable and/or complex needs virtually [79,80].

Some individuals or groups might also simply refuse to use virtual care because of negative experiences rooted in personal and/or collective histories [76]. The literature already reported discriminatory behaviors by health care providers (e.g., people of color, mental health problems, homeless people, refugees and asylum seekers, indigenous populations, LGBTQ+) [76,81,82,83,84]. Multiple barriers to using health care services for these populations have been identified: perceived discrimination, stigma, hostile attitudes, social (dis)approval, and/or embarrassment [76,81,82,83,84]. Distrust of health services is correlated with the risk of non-compliance with medical advice, prescriptions, and adherence to treatment and monitoring [76]. Nowadays, it is largely recognized that the success of a virtual consultation is highly dependent on patient engagement and self-reporting of health status and symptoms [85]. In some populations, this could result in incomplete reporting of symptoms and poor diagnoses and/or treatments [21,85]. To make things even more complicated, these same populations are also less likely to complain about the lack of, or poor quality of services and have limited power to claim their rights (e.g., self-discrimination or blame, difficulty or inability to complete complaint forms or identify services that address them) [20].

### 2.3. Data Fragmentation and Data Poverty

Quality and continuity of services is an important issue in relation to the Inverse Care Law. Pre-COVID, disadvantaged populations typically used patient portals and virtual care less, and often received health care in a fragmented way across multiple organizations (“institutional wandering”) [86,87]. Even when they were able to access and use virtual care during the pandemic, other challenges arose including “data poverty”, defined as “the inability for individuals, groups, or populations to benefit from a discovery or innovation due to insufficient [and/or quality] data” [87]. Problems of interoperability and informational discontinuity negatively affect the clinical value of EHR [10,86]. Vital information and data can be missing. Such data poverty may exacerbate existing inequalities in terms of quality and continuity of health care services.

The quality and continuity of health care services depends on the availability, quality, and integrity of data in EHR, as well as accessibility to health care providers for clinical decision-making [85]. The virtual management of complex cases (e.g., comorbidity) may thus involve compromises in the quality and continuity of health care services [88], to the detriment of disadvantaged groups.

## 3. How Does the Inverse Care Law Manifest Itself in “Direct-To-Consumer” Commercial Services?

“Direct-To-Consumer” commercial services grew considerably during the COVID-19 pandemic. This was especially the case when people were seeking alternatives to face-to-face care [10,89]. With few exceptions (e.g., when they are subcontractors for public services), these commercial platforms are for-profit businesses that are not affiliated with any particular health system or insurance plan [10,89,90]. Their services are not reimbursed by public insurance in many public and universal health systems. They are paid directly by patients (“out of pocket”) or through private insurance (e.g., through the employer) [10,91].

In the following sections, we further develop three ways in which this growing expansion of commercial platforms adds a new facet to the Inverse Care Law by opening a new profit-driven and consumerist care pipeline.

### 3.1. Consumerist and Profit-Driven Virtual Care Turns a Blind Eye to Needs

Commercial platforms are becoming popular and attractive to individuals seeking immediate and convenient access to health care services [92,93,94]. Consumerism is one of the key drivers of their increased use [95]. The primary objective of these platforms, typically backed by venture capital, is to rapidly gain market share and maximize profit [96,97]. The delivery of health care services is seen as a potentially highly lucrative investment, just like any other business opportunity. In this virtual care pipeline, market forces determine supply, and equity is not the primary concern [2,96,97]. Their target clientele would very unlikely include disadvantaged and/or underserved areas or vulnerable social groups [98]. Rather, their services are designed and delivered to homogeneous, wealthy, urban, and educated populations, possessing the necessary digital devices and a high-quality internet connection [94,99]. Due to their cultural, social, and economic capitals, these social groups are well placed to have privileged access to such services [10,93]. They are also the most able to adapt quickly to changes in services. Moreover, they tend to be healthier and more motivated to adopt healthy behavior (e.g., sport, good diet, good quality housing), thus facing fewer morbidities and risk factors. Dahlgren et al. (2021) reported that, even when they act as subcontractors to the public system, these services are more likely to be used by young people, with high education and income levels, i.e., those who are less affected by the unequal distribution of health care and services [100].

In short, the Inverse Care Law was prescient before its time, with the rise in financial barriers to accessing commercial services.

### 3.2. Episodic and Limited Services over Continuity and Comprehensiveness

Commercial providers tend to deliver a limited set of services and primarily focus on low-risk patients [20,48]. Such services are not designed to meet the needs of patients with complex needs who do not fit into a standard “15-min” consultation model focused on one problem per visit [6,9]. According to Greenhalgh et al. (2019), they are “marketed to young adults, allegedly taking this group and leaving the [health system] to care for older and sicker individuals” [101]. Such services are rarely designed to ensure continuity of care [89]. By inducing a demand for non-essential services, these platforms may shift human resources away from health systems that are already understaffed, underfunded, and ill-equipped to meet the complex needs of the population [6,48,102].

In this context, the Inverse Care Law is manifest in the fragmentation and lack of continuity and comprehensiveness of services, especially for patients with chronic diseases and co-morbidities. These are mainly disadvantaged populations. Because the services are aimed at low-risk populations, these platforms could induce an inflation of demand, including for non-essential services, which takes away the care needed by more complex patients and/or disadvantaged groups. As with communicating vessels, there is a risk that there will be even less clinical capacity to support disadvantaged populations if a significant part of the workforce offers services to commercial providers.

### 3.3. Low Prices as a Threat to the Quality and Safety of Services

Some disadvantaged people with limited resources may use “cheap” (low direct cost) platforms whose effectiveness, quality, and safety are questionable [13]. Such platforms are flourishing in a context of regulatory “laisser-faire” and policy vacuum [10,20,34,48]. Laws and regulatory frameworks are not always adapted, particularly with respect to the protection of patient data (e.g., secondary use, sales to insurance and/or pharmaceutical companies, subcontracting), as well as the control and verification of the qualifications and skills of clinicians. Those employed by these platforms may be in jurisdictions and/or countries with less stringent requirements in terms of quality and safety of care or may have experienced qualification issues in more stringent health care organizations.

In sum, the Inverse Care Law is manifest when high-need communities are exposed to low-quality healthcare and safety risks when using cheap commercial services.

## 4. Implications for Policy-Makers, Practitioners, Researchers, Public and Patients

In the previous sections, we illustrated how virtual care follows the Inverse Care Law and may add fertile ground to its expansion. Beyond geographical barriers to access and use of health care services, virtual care magnifies other considerations that compound inequalities such as socio-economic profile, digital and health literacy, age, gender, and ethnic and/or cultural identities. These dimensions influence, positively or negatively, the capacity for accessing and using digital technologies for patients to benefit from virtual care. The latter has served as a powerful reminder that inequalities are the result of economic, social, political, and even ideological dynamics rooted in society and are not simply a short-term product of COVID-19. The pandemic has only helped to increase the visibility of these dynamics, particularly through the prism of virtual care.

In line with the dominant techno-optimistic utopia, intensified by the COVID-19 crisis, certain decision-makers and technology promoters largely present virtual care as a “practically perfect” solution [10,11,21,47]. The pandemic was initially flagged as an “opportunity in a crisis” for giving virtual care the push it needed [12,57]. It was commonly assumed that widespread availability of virtual care would automatically benefit populations who face difficulties with accessing health care [98,103]. Regulatory and policy changes, including reimbursement for services and relief from legal requirements, pushed many clinicians, hospitals, clinics, and health systems to provide services in virtual form [88,104,105,106,107]. Thus, virtual care became a key pipeline for service provision, particularly for those already at a historical advantage in terms of the availability and ease of access to and use of health care services (e.g., because of clinical workforce availability). What emerged from the experience of COVID-19 is that digital technologies are in fact associated with changes that are likely to extend and entrench the Inverse Care Law in private, as well as public and universal health systems.

Decision-makers responsible for designing and delivering health care services (e.g., policy-makers, commissioners, providers, and professionals) need to acknowledge that even when reimbursed by the health system, virtual care can exacerbate existing disparities [98,103]. The rapid transition to virtual care took place when part of the population, especially marginalized or disadvantaged, did not want to, or could not engage in it. This meant that those most in need were least likely to access and use virtual services. The implications are profound, with the potential to generate what Veinot et al. (2018) referred to as “intervention-generated inequalities”. This is when an intervention disproportionately benefits the most advantaged individuals and groups and perpetuates inequalities in access to essential services for people who are already marginalized [30,108,109]. Those affected by such “digital redlining” are typically those who already experience poor health outcomes [110]. In public and universal health systems, virtual care in the context of COVID-19 has thus challenged the principle of access to health care services based on need, rather than on the ability to pay. For example, in Canada, virtual care may have breached one or more of the five foundations of the Canada Health Act: public administration, comprehensiveness, universality, portability, and accessibility [10,111].

The way virtual care has replicated and/or strengthened the Inverse Care Law offers an opportunity to initiate a critical and reflexive perspective on this service modality in health systems. To help better recognize and eventually address the deleterious effects of this law in relation to virtual care, and equity more broadly, we set out policy, practice, and research directions below (see Box 2 for an overview).

Box 2 **Key policy, practice, and research avenues for addressing the problem of the Inverse Care Law.**

**1. Key considerations for decision-making, policy, and practice:**
  - Ensuring that health professional federations and colleges, alongside other national and regional decision-makers, consider issues of inequality of access and use when planning, commissioning, and supporting digital health;
  - Access to and use of digital health services requires material and financial resources, as well as skills and knowledge that not all patients have;
  - Considering more carefully the “capabilities” of individuals and groups is vital for equitable access to and use of digital health services;
  - Ensuring that people always have the choice (and freedom) to access services physically remains foundational to contemporary health service delivery;
  - It is critical to recognize technology as a super-determinant of health: “bandwidth as a human, or a vector of, right”;
  - Social policies are needed that can facilitate access to technology and equipment, and improve the digital literacy of individuals and groups;
  - The role and place of “Direct-to-Consumer” platforms in public and universal health systems needs to be clear. These health systems should not limit their role to fixing market failures, but actively contribute to the co-creation of “public health value” through innovation;
  - The role of venture capital and digital economy companies in the trajectory of digital health technologies is essential but needs to be clear and transparent.
**2. Future research directions:**
  - Interrogate the “techno-solutionist discourse” on technology as the solution to “all the ills” of the health system;
  - Better contextualize research results on digital health technologies, enabling appreciation of the settings in which digital health “works” and potential for transfer and adoption elsewhere in the health system;
  - Examine digital technologies as a (super)-determinant of health;
  - Consider the social distribution of benefits, risks, and opportunity costs of digital health technologies;
  - Surface the unintended consequences of digital health technologies for disadvantaged patients and populations;
  - Analyze the conditions and factors for adoption and use of digital health technologies by disadvantaged patients and populations;
  - Examine the impact of venture capital on the provision of virtual care in health systems, and how it shapes them;
  - Focus more on the services, not just the technology, when considering the inclusion of disadvantaged patients and populations in research on digital health technologies.

### 4.1. Beyond Availability, Capabilities should Be at the Center of Digital Health Practices

One key take home message for virtual care designers and providers is that benefiting from virtual care requires resources, skills, and knowledge to navigate the complexity of a digitalized health system (e.g., digital access, literacy, social support, and cultural and economic capitals) [112]. It is not enough for virtual care to be made available for everyone, even when it is reimbursed by the health system. Users must be capable of benefitting from it [113].

The concept of “capabilities” draws attention to “what a person is able to do or be” [113,114]. It points out that the availability of primary goods is not enough, people should also be able to access and use them. It requires considering how social groups vary greatly in their ability to “convert” primary goods into good living [114]. In this regard, quality hardware, up-to-date software, high-speed internet, etc. are disproportionately accessible and available to wealthy, educated, digitally literate, socio-politically and culturally integrated, young and urban individuals and groups [30]. The use of technology as the main channel in the provision of health care and services could deprive some disadvantaged populations of their right and opportunity to access, but especially to use the services. This includes the ability (and freedom) to access health care services physically.

### 4.2. Addressing Digital Technologies as a Human Right Issue in Social Policies

Both before and during the pandemic, strong social policies, especially those that address the social determinants of health (e.g., housing, education, basic income, physical environment) are an important lever to promote the health and well-being of the population (e.g., “Health in All Policies”) [9,115,116,117]. The determinants of health are defined as the “conditions in which people are born, grow, live, work and age” and are “shaped by the distribution of money, power and resources” [109]. To date, social policies are still struggling to take into account and address the new challenges associated with the central role that digital technologies play in all spheres of society [118]. This is even more paradoxical as it is already established that technological innovations primarily benefit the most privileged. As a result, they can widen the socio-economic gap and increase health and social inequalities [115,119,120].

Digital technologies are now increasingly recognized as “super-determinants” of health [10,115,121]. They “open the door” to other determinants of health, which are now mainly accessible online: employment, social assistance, education, housing, family and social networks, and health care services [121]. These determinants are shaped and produced through the intersectionality of social positions and identities, and can have a negative amplifying effect on the health outcomes of individuals and groups [109]. As the “theory of fundamental causes” could explain too, differential access to and use of virtual care is related to other resources determined by the socio-political and economic status, as well as the history (e.g., stigma and discrimination) of a person or a group [30,122]. Decision-makers should thus keep in mind that disparities are rooted in structural and systemic factors and determinants that impose differential access to “flexible resources” such as economic, cultural, and social capitals (e.g., power, status, freedom) [30,122].

High dependence upon digital technologies is likely to continue and even increase after the pandemic. It is therefore necessary to approach digital technologies as a human right, like access to essential services (e.g., water, food, housing, education, medicines). Promising initiatives, legislation, and policies already exist and can serve as a lever. For example, in some countries (e.g., Estonia, France, Spain, Finland), the Internet is considered a human right or a vector of rights to ensure better digital inclusion among the population (e.g., broadband to access universal service) [38,123,124]. By placing access to Internet as a right on the same level as housing, food, and freedom, legislators would be obliged to put in place national or sub-national policy guidelines, and measures to address, directly or indirectly, health inequalities [38]. Such requirements can mean facilitating access to high-speed internet, quality digital tools, and equipment, as well as the acquisition of the necessary digital capabilities to take full advantage of the technology (e.g., public education programmes through community organizations).

### 4.3. Addressing Health Equity in the Digital Economy

Decision-makers should recognize that digital transformation in the health system is embedded and negotiated through broad socio-political, ideological, symbolic, and economic processes [125]. Their trajectories and evolutions are dependent on interactions where decision-makers need to be equipped to handle complex socio-political, economic, and institutional dynamics, and professional pressures [126]. In this regard, health systems are increasingly interacting, and at times in a confusing manner, with the digital economy driven mainly by venture capital [125]. “Direct-To-Consumer” platforms are here to stay because of growing consumerism [95]. These services meet the expectations of certain groups of the population who have the financial means and ability to “do without” public health services [10,91]. They are thus a prime target for market- and investment-oriented actors seeking maximum return on their investment. In public and universal health systems, these services are proliferating in a regulatory vacuum, even if they challenge principles of universality and equal access to health care services. Decision-makers should place more emphasis on the implications to population health when access is conditioned by the ability to pay and that such commercial services are not aimed at fostering health equity.

The risk for publicly funded health systems is to limit their policies to fixing market failures and possible drifts, whereas they should more than ever become active actors in the co-creation of “public health value” through innovation [127]. With the current global trends, digital health technologies may become “trapped” in development paths steered by profit and economic gains, without any real added value for health systems and society [125]. Paradoxically, if the state acts in accordance with the market, it could be complicit in the exacerbation of the Inverse Care Law and, ultimately, inequalities [128]. According to Hart (2001), “no market will ever shift corporate investment from where it is most profitable to where it is most needed” [129]. In other words, virtual care cannot be left to market forces alone. As Mazzucato points out: “making public value better justified, appreciated and evaluated would potentially open a new vocabulary for policy-makers. Rather than being mere ‘regulators’ of health care or the digital agenda, as co-creators of that care and digital transformation policy-makers would have a more justifiable right to make sure that the benefits are accessible to all” [127,130].

### 4.4. Throwing Away the Dominant Digital Health Innovation Research Paradigm

Wherton et al. (2021) reported that pre-pandemic studies on the effectiveness, acceptability, and efficiency of virtual care mainly relayed a positive message, while being potentially misleading [57]. Many studies initiated by local champions/enthusiasts, usually small and therefore lacking in power to test their central hypothesis, reported results on low-risk and digitally confident patients selected from a more diverse general population. Unsurprisingly, the results generally show that these patients were no less satisfied, did not do worse clinically, and costs were more or less similar [57]. Along decision-making and business circles, research has historically emphasized effectiveness and efficiency over equity, particularly by relying on analytical approaches that “measure averages rather than social distributions” of the benefits and risks of technologies [112]. This is while a service can produce an average population-level improvement through its beneficial effects on a large segment of the population and, concurrently, exacerbate disparities within the most disadvantaged groups [30]. This distorted view impedes the production of knowledge on what really matters in health care services, including contextual factors and conditions that influence access to and use of technologies [70]. It has been driven by a process wherein “technosolutionist experts” dominate the construction of the narrative around technology and establish “rhetorical closure”. This means that a given technological “solution is supposed to have reached stabilization, being already well defined, ready to use and able to solve the problem it sets out to solve” [131]. Such a paradigm has largely influenced the type of technologies and the population-level consequences seen so far [118]. As a result, reports and assessments emanating from such research and policies rarely, if ever, refer directly or indirectly to the Inverse Care Law and other systemic and structural barriers to health care services [6].

The COVID-19 pandemic constitutes a unique opportunity to question research on digital health technologies more broadly. As argued in this paper, there is a large gap to be addressed in terms of knowledge about the multiple impacts of virtual care and their translation into the Inverse Care Law. Further research could focus more on identifying the technologies, but also the conditions and factors that actually enable equity in virtual care access and use or, to the contrary, induce risk and exacerbate disparities [10,38]. For example, more attention should be paid to the social distribution of the opportunity costs of virtual care, particularly in view of the sacrifices made on other interventions not provided to the population [112,132]. It is essential to analyze the overall access and use of virtual care in relation to the characteristics of the populations that actually benefit and those who are excluded [71]. This helps particularly to know whether and how digital technologies affect, in practice, health inequalities and whether they confer comparable social outcomes across different population subgroups [30,76]. More attention should be given to the factors of engagement and use of digital technologies by different categories of the population, the underlying contributing conditions, and their impact on access to and use of health care services [38]. Scholars should also consider more carefully the structural and systemic contexts that influence access to and use of digital technologies and the inequalities that may be associated with them. Making a “social autopsy” of the deployment and use of virtual care in the context of COVID-19 under the prism of the Inverse Care Law is warranted [113,114,133]. This is the first step towards greater consideration of equity in digital health research and decision-making.

## 5. Conclusions

Virtual care has saved lives during the pandemic. However, there are clear and troubling signs that it has not benefited everyone equally and, in some cases, even harmed some disadvantaged individuals and groups. The Inverse Care Law offers a lens through which one can critically analyze the access, use, quality, continuity, and impact of digital technologies in the delivery of health care services. It underscores the importance of using equity as a compass to guide digital health practices. This is critical as we shift into post-pandemic times where virtual care is likely to continue to play a key role in policies and actions aimed at modernizing health systems. Although a decrease in its use has been observed in the post-crisis context, policy-makers, commissioners, providers, and professionals need to be more vigilant in developing and implementing decisive policies and actions to safeguard access, use, quality, and continuity of health care services—virtually or otherwise—for those who need it most.

## Data Availability

All data is from public articles and documents. Sources are cited in the references. The authors are available to answer any reasonable request related to the article’s content if necessary.
